# Translational Molecular Imaging Tool of Vulnerable Carotid Plaque: Evaluate Effects of Statin Therapy on Plaque Inflammation and American Heart Association–Defined Risk Levels in Cuff-Implanted Apolipoprotein E–Deficient Mice

**DOI:** 10.1007/s12975-022-01114-4

**Published:** 2022-12-09

**Authors:** Joyce M. S. Chan, Sung-Jin Park, Michael Ng, Way Cherng Chen, Wan Ying Chan, Kishore Bhakoo, Tze Tec Chong

**Affiliations:** 1 Translational Cardiovascular Imaging Group, Institute of Bioengineering and Bioimaging (IBB), Agency for Science, Technology and Research (A*STAR), 11 Biopolis Way, #02-02 , Singapore, 138667 Helios Singapore; 2 Department of Vascular Surgery, Singapore General Hospital, SingHealth, Outram Road, Singapore, 169608 Singapore; 3 Lee Kong Chian School of Medicine, Nanyang Technological University, 50 Nanyang Avenue, Singapore, 639798 Singapore; 4 Bruker Singapore Pte. Ltd, 30 Biopolis Street, #09-01, Singapore, 138671 Matrix Singapore; 5 Division of Oncologic Imaging, National Cancer Centre, Singapore, Singapore; 6 Institute of Bioengineering and Bioimaging (IBB), Agency for Science, Technology and Research (A*STAR), 11 Biopolis Way, #02-02, Singapore, 138667 Helios Singapore

**Keywords:** Atherosclerosis, Stroke, Inflammation, Molecular imaging, Statin, American Heart Association classification of plaques

## Abstract

Identification of high-risk carotid plaques in asymptomatic patients remains a challenging but crucial step in stroke prevention. The challenge is to accurately monitor the development of high-risk carotid plaques and promptly identify patients, who are unresponsive to best medical therapy, and hence targeted for carotid surgical interventions to prevent stroke. Inflammation is a key operator in destabilisation of plaques prior to clinical sequelae. Currently, there is a lack of imaging tool in routine clinical practice, which allows assessment of inflammatory activity within the atherosclerotic plaque. Herein, we have used a periarterial cuff to generate a progressive carotid atherosclerosis model in apolipoprotein E–deficient mice. This model produced clinically relevant plaques with different levels of risk, fulfilling American Heart Association (AHA) classification, at specific timepoints and locations, along the same carotid artery. Exploiting this platform, we have developed smart molecular magnetic resonance imaging (MRI) probes consisting of dual-targeted microparticles of iron oxide (DT-MPIO) against VCAM-1 and P-selectin, to evaluate the anti-inflammatory effect of statin therapy on progressive carotid atherosclerosis. We demonstrated that in vivo DT-MPIO-enhanced MRI can (i) quantitatively track plaque inflammation from early to advanced stage; (ii) identify and characterise high-risk inflamed, vulnerable plaques; and (iii) monitor the response to statin therapy longitudinally. Moreover, this molecular imaging–defined therapeutic response was validated using AHA classification of human plaques, a clinically relevant parameter, approximating the clinical translation of this tool. Further development and translation of this molecular imaging tool into the clinical arena may potentially facilitate more accurate risk stratification, permitting timely identification of the high-risk patients for prophylactic carotid intervention, affording early opportunities for stroke prevention in the future.

**Supplementary Information:**

The online version contains supplementary material available at 10.1007/s12975-022-01114-4.

## Introduction

Stroke is the second leading cause of global mortality and disability [1, 2]. Inflammation is recognised as a key driver in atherogenesis, plaque instability, and subsequent embolisation causing stroke [3]. Currently the criterion for intervention in carotid atherosclerosis is predicated on luminal diameter reduction in angiography, but it is recognised that degree of stenosis alone does not have high predictive value of stroke risk. Supporting this, the Asymptomatic Carotid Surgery Trial (ACST) has revealed that conventional clinical angiography failed to detect a high-risk asymptomatic subgroup bearing the vulnerable inflamed plaques [4].

Statins, along with their primary cholesterol-lowering benefits, have demonstrated the attenuation of systemic inflammatory markers [5, 6] and local plaque inflammation in patients [7]. Monitoring lipid level solely is inadequate for evaluating the therapeutic effect of statin, as it does not specifically report the inflammatory activities within the plaques that were attenuated by this therapy. Hence, a wide range of molecular imaging tools have emerged to directly interrogate the change in these biological processes in local plaques after a therapeutic intervention, not captured by conventional anatomical imaging or serological tests [8–13].

^18^F-fluorodeoxyglucose positron emission tomography (^18^F-FDG PET) integrated with computed tomography (CT) is a promising tool to differentiate benign from vulnerable plaques [11, 14], predict cardiovascular prognosis [15, 16], and monitor anti-inflammatory effects of statins [10, 12, 17]. However, the need for specialised PET/CT facilities and the risk of radiation may hamper the routine clinical use of this imaging tool in cardiovascular disease evaluation [18]. Contrast-enhanced ultrasound (CEUS) using microbubbles as intravascular probes have been utilised to detect intraplaque neovascularisation and inflammation in human carotid plaques [19, 20], and to track the response to statins therapy in animals [21]. Carotid MRI has demonstrated in vivo characterisation of lipid-rich necrotic core and intraplaque haemorrhage, both of which are high-risk plaque features and strong risk predictors for cerebrovascular events [22–24]. Despite vast advancement in plaque MRI techniques, molecular MRI has further enabled interrogation of the underlying inflammatory processes within atherosclerotic plaques in pre-clinical and clinical studies [7, 25–27]. Nonetheless, there is still no imaging tool in the *routine* clinical practice to evaluate the inflammatory status within atherosclerotic plaques.

Atherosclerosis is initiated by the elevated expression of adhesion molecules on the activated endothelium [3]. These pathophysiologically “inducible” adhesion molecules, namely vascular cell adhesion molecules-1 (VCAM-1; CD106) and P-selectin (CD62P), play vital roles in the monocyte recruitment into vascular tissues in this inflammatory process [28]. The interplay between firm adhesion and rolling mediated by VCAM-1 and selectins demonstrated a synergistic binding effect between the monocytes and activated endothelium [29]. Additionally, VCAM-1 is expressed on the primary plaque components, namely activated macrophages and smooth muscle cells [30]. Based on this, dual-ligand iron particles targeting both VCAM-1 and selectins were constructed and shown to achieve more efficient binding, than targeting with either single-ligand in our work on patient carotid plaques [31], as well as earlier studies [32–34]. Exploiting the vital functions of these adhesion molecules in atherosclerosis, their tightly regulated spatio-temporal expression, and ready availability via the bloodstream warrant them as valued targets for molecular imaging [28].

Building on previous studies [32, 35, 36], we have used a periarterial cuff to generate a progressive carotid atherosclerosis model in apolipoprotein E–deficient mice. This model produced clinically relevant plaques with different levels of risk, fulfilling American Heart Association (AHA) classification, at specific timepoints and locations, along the same carotid artery [35]. Exploiting this platform, we have established a molecular magnetic resonance imaging (MRI) tool utilising dual-targeted microparticles of iron oxide (DT-MPIO) against VCAM-1 and P-selectin, as a smart MRI probe, to directly report the inflammatory status in local plaques for characterisation and risk stratification of carotid atherosclerotic disease. We sought to ascertain the capability of in vivo DT-MPIO-enhanced MRI tool to (i) target and discriminate the high-risk vulnerable carotid plaques from stable plaques, (ii) quantitatively track the inflammatory status of plaque as it progresses from early to advanced disease stage, and (iii) monitor the anti-inflammatory effect of statin therapy longitudinally in the cuff implantation progressive atherosclerosis model. Furthermore, we have used clinically relevant parameters, including AHA classification of human plaques, to evaluate the DT-MPIO-defined therapeutic response in atherosclerosis, approximating the clinical translation of this molecular imaging tool.

## Material and Methods

### Experimental Design, Cuff-Implanted Animal Model, and Statin Treatment

**Fig. 1:**
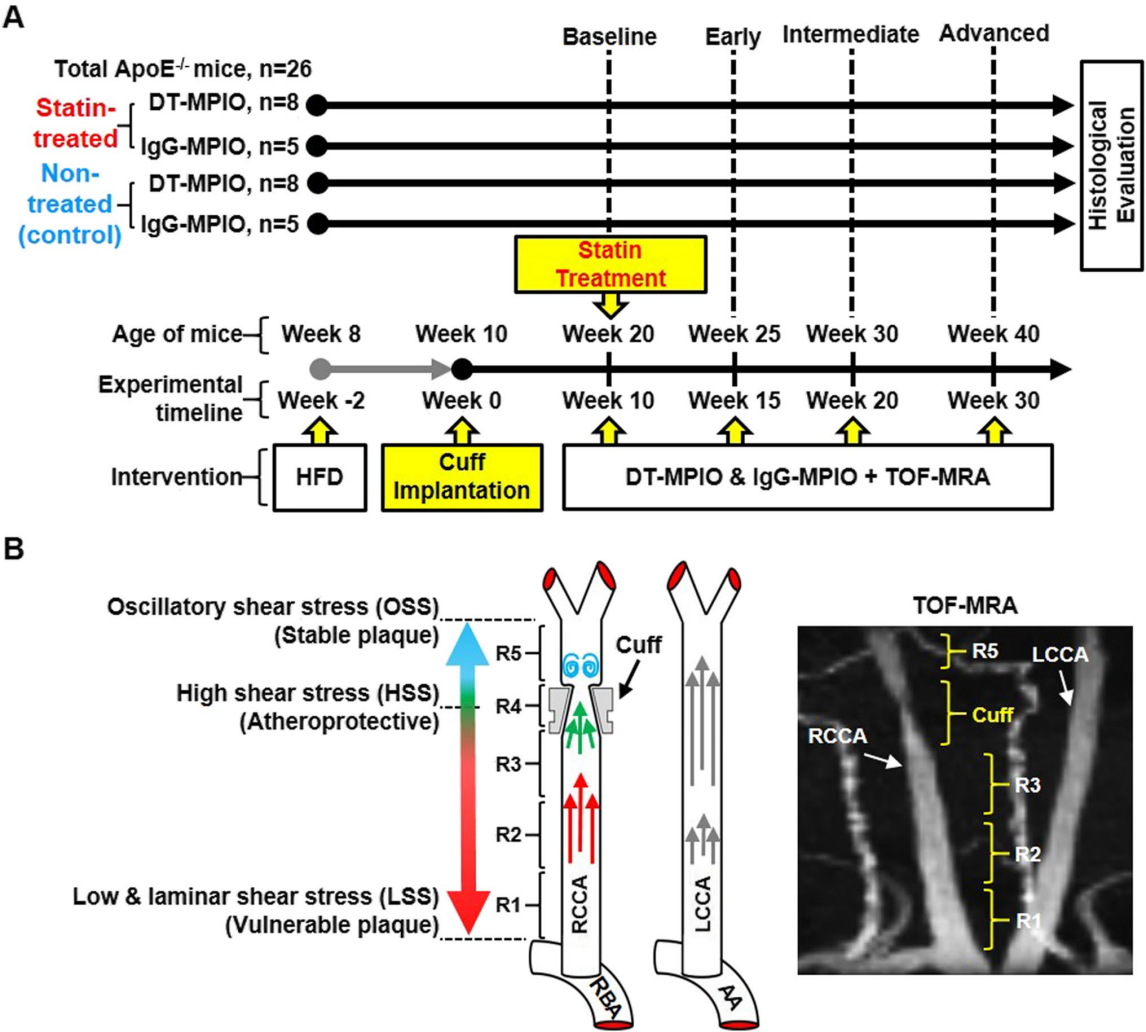
(A) Experimental design. ApoE^−/−^ mice (DT-MPIO statin-treated group: *n* = 8; DT-MPIO non-treated group: *n* = 8; control IgG-MPIO statin-treated group: *n* = 5; control IgG-MPIO non-treated group: *n* = 5) were used to develop the atherosclerosis model as reported [35]. Mice were first put on high fat diet (HFD). Cuff implantation surgery on the RCCA was then performed on all animals at week 0 (experimental timeline), and LCCAs were untreated as internal control. Statin treatment was started on both statin-treated groups at week 10. DT/IgG-MPIO-enhanced TOF-MRA was performed on all groups at week 10 (baseline), week 15 (early), week 20 (intermediate), and week 30 (advanced). Histological evaluation was performed after the final MRI time-point (week 30). (B) Schematic diagram of the periarterial cuff with corresponding TOF-MRA image. A tapered end shear-stress modifying cuff was implanted in the RCCA, creating distinctive zones of LSS in upstream, HSS within cuff and OSS in downstream of the implanted carotid artery. The different forms of shear-stress lead to differential formation of vulnerable, inflamed plaques (LSS) and stable plaques (OSS), as well as disease-free zone (HSS). In the TOF-MRA image, the distinct regions (i.e. R1, R2, R3, R4 (cuff), and R5) were depicted in RCCA. No cuff was implanted on the LCCA

The experimental design is illustrated in Fig. 1A. Apolipoprotein E (ApoE) knock-out (ApoE^−/−^) mice (Taconic Biosciences) were utilised to create the cuff-implanted atherosclerosis model. The surgery for implanting a shear-stress modifying cuff (Promolding BV, Netherlands) on carotid artery has been described in previous work [32, 34–37]. Distinctive zones of low shear stress (LSS), high shear stress (HSS), and oscillatory shear stress (OSS) were generated in the upstream, within cuff, and downstream of the implanted carotid artery, respectively, by means of this tapered end cuff (Fig. 1B). Previous studies reported that these different forms of shear stress contribute to the differential formation of vulnerable, inflamed plaques (LSS), and stable plaques (OSS), as well as disease-free zone (HSS) [32, 34–37].

To examine the longitudinal effects of statin treatment on atherosclerotic plaques, 4 groups of ApoE^−/−^ mice were used (DT-MPIO statin-treated group: *n* = 8; DT-MPIO non-treated group: *n* = 8; control IgG-MPIO statin-treated group: *n* = 5; control IgG-MPIO non-treated group: *n* = 5), where IgG-MPIO was used as a control to evaluate the non-specific binding of particles to the plaques. At 8 weeks of age, high-fat diet (Altromin C1061) was started on all mice and maintained throughout the study. At 10 weeks of age, the cuff was surgically implanted on the right common carotid artery (RCCA), leaving left common carotid artery (LCCA) untreated as internal control [32, 34, 35]. From 20 weeks of age, one dose of 50 mg/kg simvastatin in 0.5% carboxymethyl cellulose was orally administrated per day for 20 weeks in the treatment groups, while no statin was administered in the control groups. All groups were followed up longitudinally by serial in vivo MRI and euthanised at the end of the final MRI scan for histological analysis. All procedures involving animals were under an approved protocol with the A*STAR Institutional Animal Care and Use Committee (#191,459).

### In Vivo* MRI of Carotid Arteries*

All groups underwent DT-MPIO-enhanced MR imaging of carotid arteries at 10 (baseline, before commencement of statin), 15, 20, and 30 weeks post-cuff implantation for risk stratification [34] and longitudinal tracking of atherosclerotic plaques [34]. All in vivo MRI was performed on an 11.7-T MRI (Bruker, BioSpec), fitted with a 40-mm mouse-body coil (Bruker). Mice were anaesthetised using 1.5 to 2% isoflurane. Time-of-flight angiography (TOF-MRA) was done with a three-dimensional (3D) fast low-angle shot (FLASH) gradient-echo sequence to obtain a pre-contrast scan: field-of-view (FOV) = 24 × 24 × 10 mm; acquisition matrix = 256 × 256 × 64; TR = 12.0 ms; TE = 2 ms; NA = 4; flip angle = 20°; slab thickness = 10 mm; scan time = 10 m 54 s. After completion of pre-contrast MRA, DT-MPIO (*n* = 16) or IgG-MPIO (*n* = 10), 30 mg Fe/kg, was injected intravenously via a catheter. The previously applied angiography sequence was repeated up to 2 h post-contrast.

### MR Image Analysis

Dataviewer (Bruker) and CTan (Bruker) imaging software were used to process MR images as previously published [34]. Briefly, using Dataviewer, RCCA and LCCA images for each mouse was re-aligned into the cranio-caudal position. After which, the post-contrast carotid with largest hypointense signal was registered to the pre-contrast reference image. Using CTan, the carotid artery was split into 64 axial cross-sectional slices and assigned to the following regions, R1–3: slices from brachiocephalic branch to proximal end of the cuff, equally divided into 3 regions; R4: slices within the cuff (i.e. from proximal end to distal end of the cuff); R5: slices from the distal end of the cuff to carotid bifurcation. The axial slices of carotid vessels were delineated by auto-selection at a predefined (default) threshold using the CTan analysis software. The predefined threshold checked that all pixels with intensities above the threshold limit were turned on, while all other pixels were turned off, converting the grey-scale images into binary images. The threshold was first applied on the control carotid (LCCA) and was examined to ensure accuracy. The selected threshold was then performed on all carotids (both RCCA and LCCA) in the same scan. The degree of change in dark signal attributed by MPIO was calculated using the difference in area under curve (AUC) between pre- and post-contrast graphs. For both the pre- and post-contrast images, the carotid area in each slice was calculated using the total number of white pixels in each slice and a curve (graph plot) of carotid area versus slice number was plotted. From the paired curve (pre-/post-contrast), the magnitude of change in hypointense signal between the pre- and post-contrast carotid image, induced by MPIO, was quantified by the difference in area under the curve (AUC) and represented as Fig. 3C. A simplified diagram to illustrate the quantification of MPIO-induced hypointense signal by the difference in AUC is included (Supplementary Fig. 1).

### Statistical Analysis

The relationship between the change in MR signal and fluorescent intensity signal measured in distinct locations of carotid artery was represented by box and whisker plots. Average intensity values of DT-MPIO, oil red O, and IHC signals (MOMA-2, CD62P, VCAM-1, and SMA) in each zone of the carotid (i.e. R1, 2, 3, and 5) per animal were presented. Student’s *t*-tests were performed amongst R1, 2, 3, and 5. In addition, relationship between the change in MR signal and the following groups was examined: (i) DT-MPIO fluorescent signal, (ii) individual biomarker expression (oil red O, MOMA-2, CD62P, VCAM-1, and SMA). R1, 2, 3, and 5 samples were used. R4 was excluded from analysis as it was recognised as a disease-free athero-protective region. *p* < 0.05 was regarded as statistically significant.

Vulnerability index score was calculated as previously reported [35, 38]. Briefly, the percentage of macrophage (MOMA-2 immuno-reactivity) and necrotic core filled plaque area was divided by the percentage of smooth muscle cells (SMA immuno-reactivity) and collagen-filled plaque area. A high index score indicates high plaque vulnerability.

Detailed methods for the synthesis of fluorescent-labelled DT-MPIO and evaluation of histological samples are shown in the Supplementary Methods.

## Results

### In Vivo* DT-MPIO-Enhanced MRI: Monitoring Response to Statin Longitudinally in All Regions of RCCA at All Disease Stages*

**Fig. 2:**
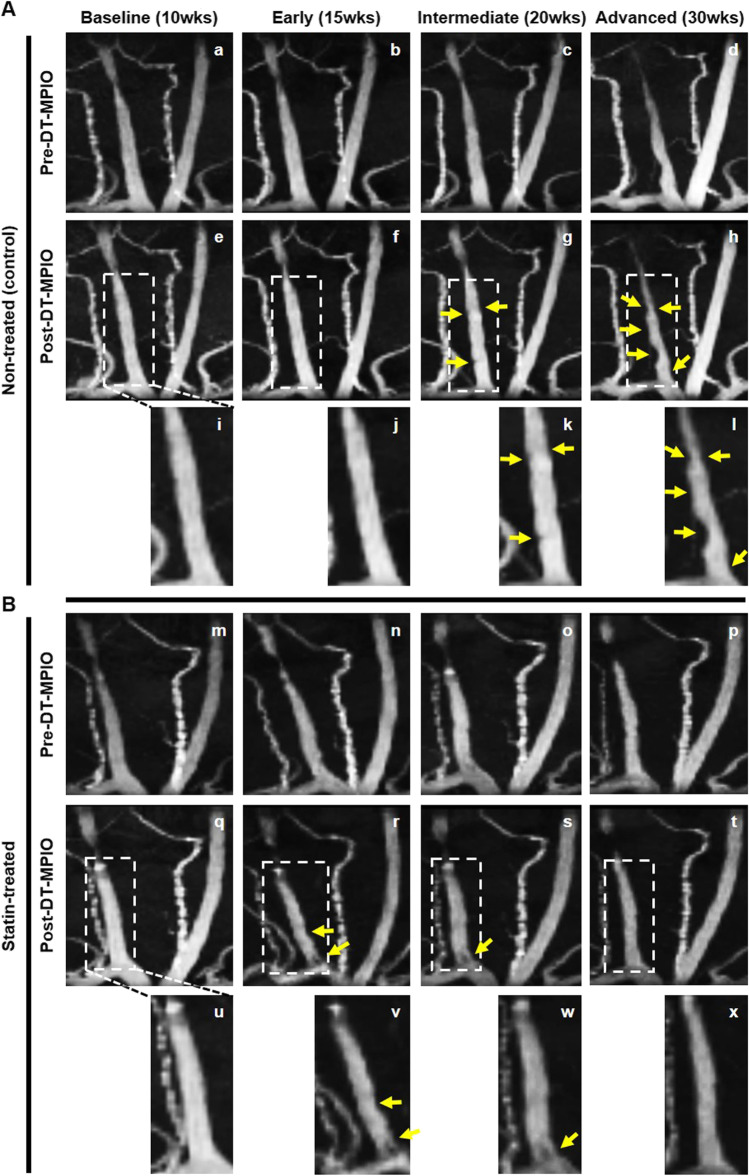
Serial in vivo DT-MPIO-enhanced MRA of carotid arteries to monitor response to statin treatment longitudinally. (A) DT-MPIO non-treated group. (a–d) Pre-DT-MPIO MRA images of carotid arteries as atherosclerosis progresses from baseline (10 weeks) to early (15 weeks), intermediate (20 weeks), and advanced (30 weeks) stage. (e–h) Post-DT-MPIO MRA images. (i–l) Magnified MRA images of post-DT-MPIO RCCA. No new dark signals were identified in baseline and early stages (i and j); small amount of new signals (yellow arrows) in intermediate stage (k) and new conspicuous hypointense dark signals (yellow arrows) in advanced stage were detected in R1 and R2 (l). (B) DT-MPIO statin-treated group. (m–p) Pre-DT-MPIO MRA images of carotid arteries as atherosclerosis progresses from baseline to early, intermediate, and advanced stage. (q–t) Post-DT-MPIO MRA images. (u–x) Magnified MRA images of post-DT-MPIO RCCA. No signal was observed in baseline before commencement of statin; new mild dark signals (yellow arrows) were detected in early and intermediate stages (v and w), while no new signal in advanced stage was identified in R1 and R2 (x)

In the DT-MPIO non-treated group, no new discrete hypointense signal was identified in either baseline or early stages; small amount of new signal in intermediate stage and new conspicuous hypointense dark signals in advanced stage were detected in R1 and R2 of RCCA post-DT-MPIO injection (Fig. 2A a–l). A new dark signal in the post-contrast image is defined by the appearance of a fresh hypointense (dark/black) area located in the lumen of the carotid artery, which was previously not seen, or noticeably larger than that in the pre-contrast image. In the DT-MPIO statin-treated group, no signal was observed in baseline before commencement of statin; a new mild dark signal was detected in early and intermediate stages, while no new signal in advanced stage was identified in R1 and R2 of RCCA in post-contrast images (Fig. 2B m–x). Conversely, in both IgG-MPIO statin-treated and non-treated groups, no new distinct hypointense signal was identified throughout all disease stages of post-IgG-MPIO images (Supplementary Fig. 2).

**Fig. 3:**
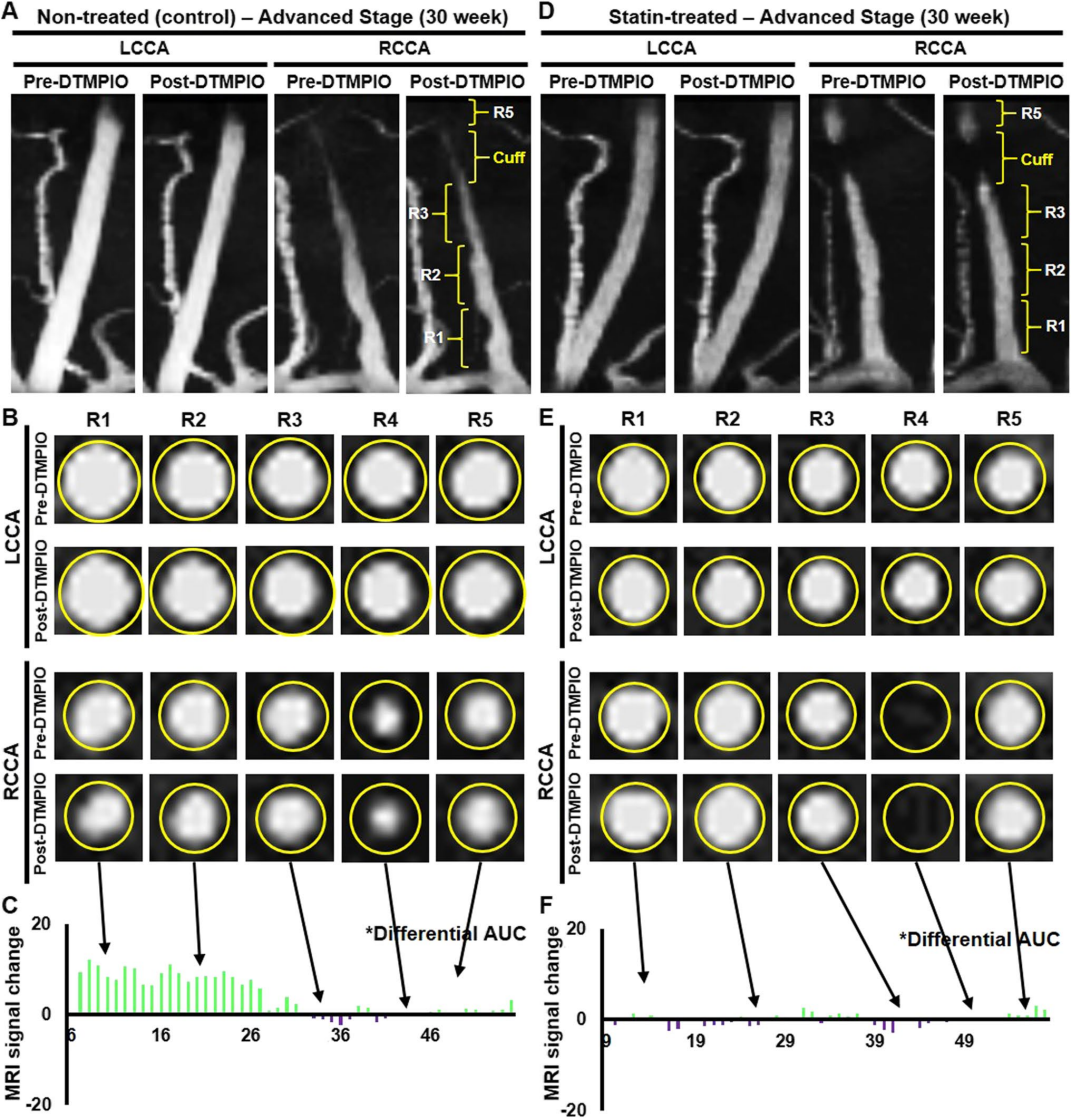
Quantification of DT-MPIO-induced MR signal change in statin-treated and non-treated groups. (A) Representative DT-MPIO-enhanced TOF-MRA images of carotid arteries in non-treated group at 30 weeks. New discrete dark signals detected in R1-2 of RCCA in post-contrast image. (B) Corresponding transverse plane MR images of LCCA and RCCA in non-treated group. New discrete dark signals detected on the matching post-DT-MPIO transverse planes in R1-2 of RCCA. (C) Quantification of MRI signal change between pre- and post-contrast MR images of carotid arteries. The degree of change in MR signal was higher in R1 and R2 of RCCA than in R3, R4, or R5. (D) Representative DT-MPIO-enhanced TOF-MRA images of carotid arteries in statin-treated group at 30 weeks. No new dark signal detected in post-contrast images of both carotid arteries. (E) Corresponding transverse plane MR images of LCCA and RCCA in statin-treated group. No new discrete dark signal detected on the matching post-DT-MPIO transverse planes in both carotid arteries. (F) Quantification of MRI signal change between pre- and post-contrast MR images of carotid arteries. The degree of change in signal was minimal in the whole RCCA

Concurring with 3D-TOF angiography at 30 weeks (Fig. 3A), new prominent dark signals were detected on the matching transverse planes in R1-2 of RCCA in the DT-MPIO non-treated group (Fig. 3B). Conversely, no new dark signal was identified on the transverse planes in RCCA regions (i) R3, (ii) R4 or (iii) R5, and (iv) whole LCCA (Fig. 3B). Supporting this, the degree of change in MR signal was higher in RCCA regions R1 and R2 than (i) R3, (ii) R4 or (iii) R5 (Fig. 3C), and (iv) whole LCCA in the post-DT-MPIO images. In the DT-MPIO statin-treated group, no new dark signal was identified on 3D-TOF angiographic images (Fig. 3D) and the respective transverse planes in all regions (R1–5) of RCCA and LCCA in post-DT-MPIO images (Fig. 3E). The degree of change in signal was minimal in the whole RCCA (Fig. 3F) and LCCA after contrast administration. In both IgG-MPIO statin-treated and non-treated groups, no new distinct dark signal was observed on 3D-TOF angiography (Supplementary Fig. 3A and D) and the transverse planes of post-contrast images throughout RCCA and LCCA (Supplementary Fig. 3B and E). Supporting this, the degree of change in signal, induced by IgG-MPIO, remained minimal throughout RCCA (Supplementary Fig. 3C and F) and LCCA.

### Quantitative Analysis of DT-MPIO-Induced MR Signal on Response to Statin at All Disease Stages

**Fig. 4:**
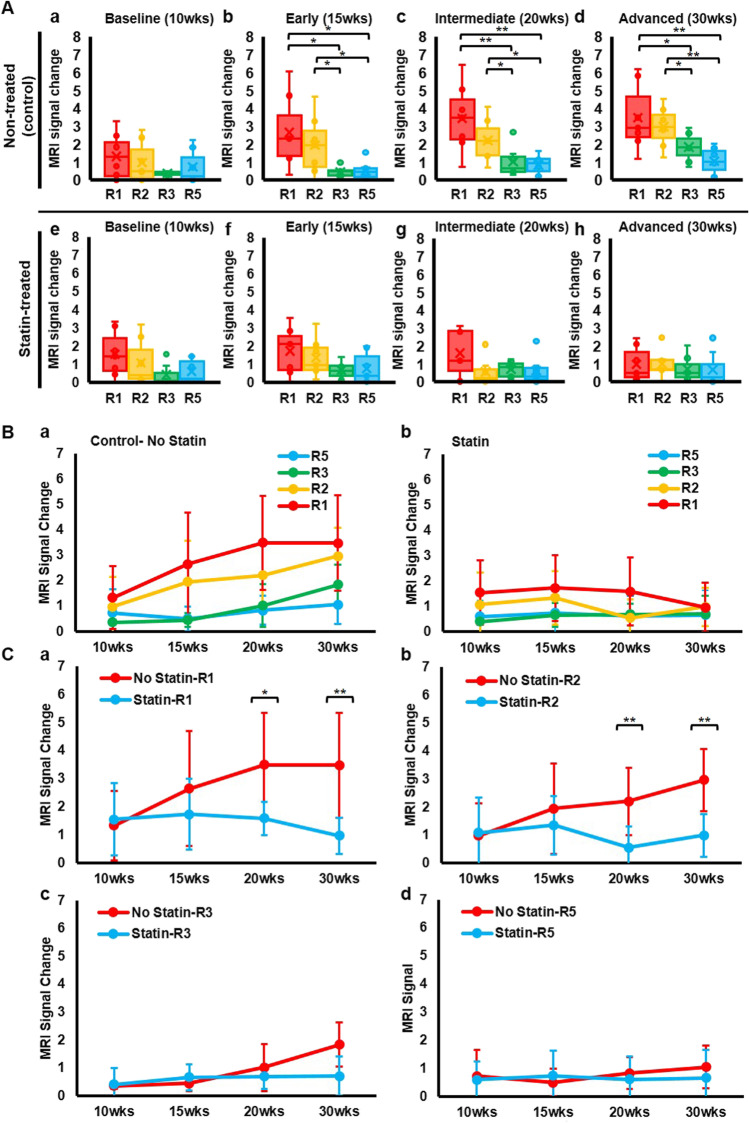
DT-MPIO-induced MR signal change in different regions of RCCA at all disease stages in statin-treated and non-treated group. (A) MRI signal change in different regions of RCCA at each disease stage. (a–d) Non-treated group. No significant difference in the degree of change in MR signal detected in all regions in the baseline. MR signal change was significantly greater in R1 and R2 than that in R3 and R5 in early, intermediate, and advanced stages. (e–h) Statin-treated group. No significant difference in the MR signal change detected in all regions at all disease stages. (B) MRI signal change in different regions of RCCA as disease progresses. (a) Non-treated group. No significant difference in the degree of change in MR signal detected in all regions in the baseline (10 weeks). As disease progresses, the MR signal change was significantly greater in R1 and R2 than that in R3 and R5 in early (15 weeks), intermediate (20 weeks), and advanced (30 weeks) stages. *p* values are indicated in A b, c, and d. (b) Statin-treated group. No significant difference in the MR signal change detected in all regions throughout all disease stages. (C) MRI signal change at each region of RCCA as disease progresses, between non-treated and statin-treated groups. (a) MR signal change induced in R1 plaques of the non-treated group was significantly greater compared to those in the statin-treated group as disease progresses to intermediate (20 weeks) (p* < 0.05) and advanced (30 weeks) (p** < 0.01) stages. (b) MR signal change induced in R2 plaques of the non-treated group was significantly greater compared to those in the statin-treated group as disease progresses to intermediate (20 weeks) (p** < 0.01) and advanced (30 weeks) (p** < 0.01) stages. (c–d) No significant signal change in R3 and R5 plaques was detected between non-treated group and statin-treated group in all disease stages. All groups without * or ** had no significant differences detected

Furthermore, we examined the degree of change in MR signal, induced by DT-MPIO, in distinct regions of RCCA at all disease stages in both statin-treated and non-treated groups (Fig. 4). In the non-treated group, no significant difference in the degree of change in MR signal was detected in all regions in the baseline (10 weeks after cuff implantation) (Fig. 4A and Ba). As atherosclerosis progresses, the MR signal change was significantly greater in R1 and R2 than that in R3 and R5 in early (Fig. 4Ab), intermediate (Fig. 4A c), and advanced (Fig. 4A d) stages (Fig. 4B a). By contrast, in the statin-treated group, no significant difference in the MR signal change was detected in all regions at all disease stages (i.e. baseline (Fig. 4A e), early (Fig. 4A f), intermediate (Fig. 4A g), and advanced (Fig. 4A h) (Fig. 4B b).

A comparison between non-treated and statin-treated groups shows that, in the intermediate and advanced stages of atherosclerosis progression, the MR signal change induced in R1 and R2 plaques of the non-treated group was significantly greater compared to those in the statin-treated group (Fig. 4C a and b). However, no significant signal change in the plaques in R3 and R5 was detected between the non-treated group and statin-treated group in all disease stages (Fig. 4C c and d).

### Quantitative Analysis of Plaque Inflammation and Vulnerability on Response to Statin

**Fig. 5:**
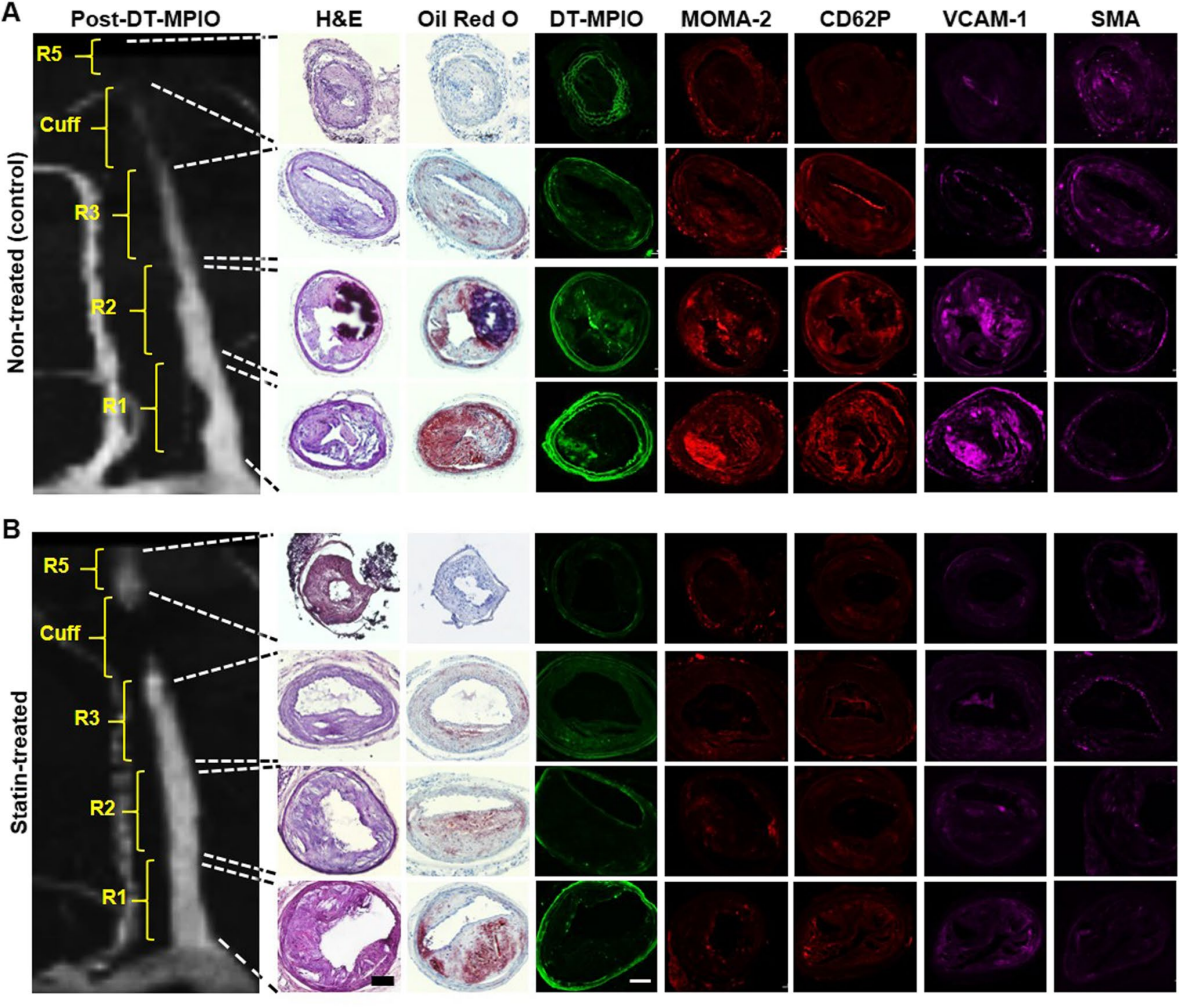
Histological analysis of all regions in RCCA in DT-MPIO statin-treated group and non-treated group. (A) Representative post-DT-MPIO MRA image of RCCA in non-treated group with matching histological sections. Substantial quantity of fluorescent-tagged DT-MPIO was identified in R1 and R2 plaques. These plaques display high-risk inflamed plaque phenotype (i.e. increased level of inflammation biomarkers: MOMA-2, P-selectin, VCAM-1, large “destabilising” lipid burden (oil red O), low “stabilising” smooth muscle cells (SMC) content in the intima). Small amount of DT-MPIO observed in R3 plaques with moderate levels of inflammation and lipid burden. Minimal amount of DT-MPIO observed in R5 plaques, which bear fairly stable and less inflamed phenotype (i.e. reduced level of inflammation biomarkers: MOMA-2, P-selectin, VCAM-1, small lipid burden, and higher SMCs content)). (B) Representative post-DT-MPIO MRA image of RCCA in statin-treated group with matching histological sections. Minimal amount of DT-MPIO was observed in R1, R2, R3, and R5 plaques. The plaques display reduced levels of inflammation biomarkers (i.e. MOMA-2, P-selectin, VCAM-1) and lower level of “destabilising” lipid content as compared to those in the corresponding regions in the non-treated group. However, the “stabilising” SMC amount in R3 and R5 plaques were also lower than those in the non-treated group

**Fig. 6:**
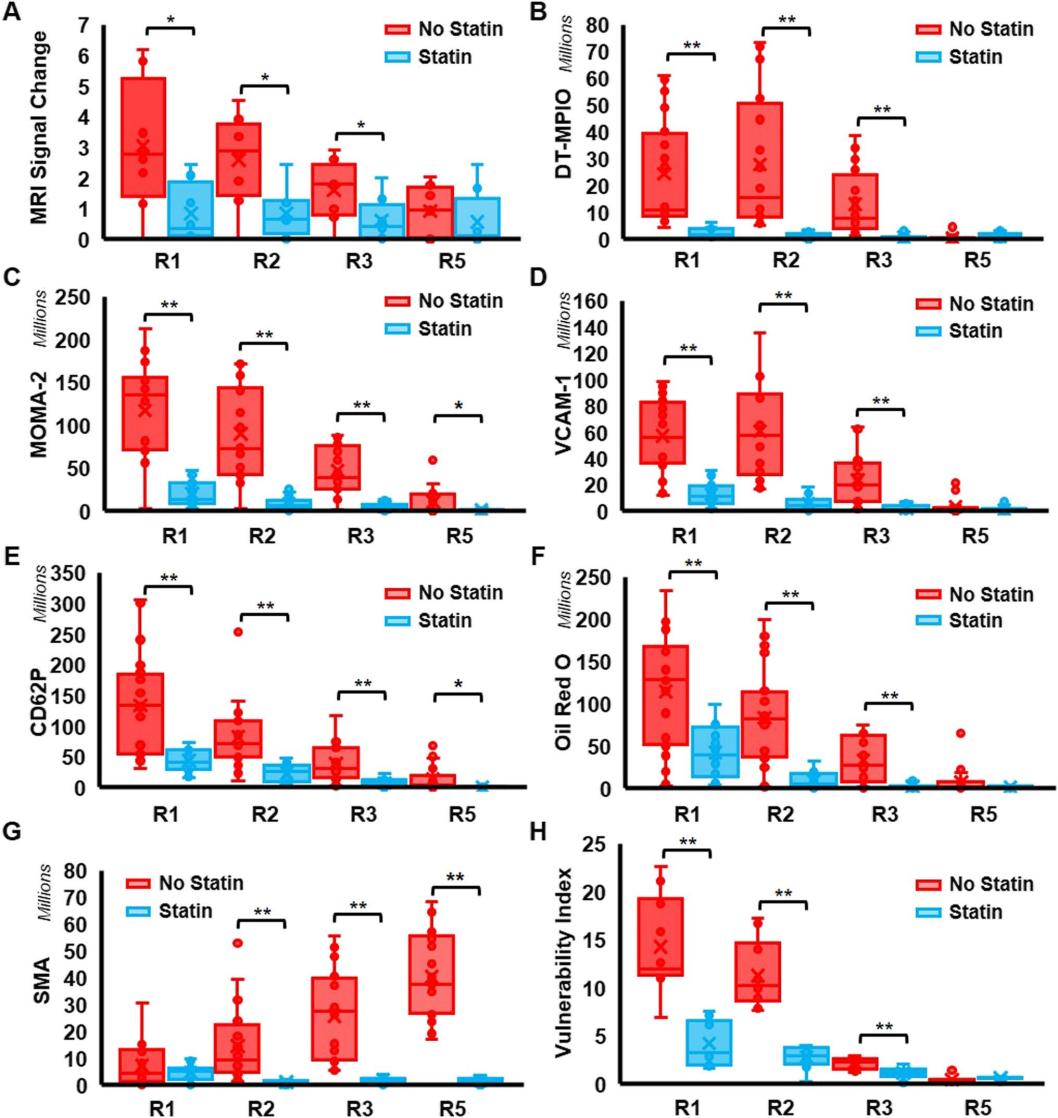
Quantitative analysis of plaque inflammation and vulnerability in statin-treated and non-treated groups. (A) MRI signal change between pre- and post-DT-MPIO MRA images. The degree of change in MRI signal in R1, R2, and R3 plaques of RCCA was significantly higher in the non-treated group than in the statin-treated group (p* < 0.05). (B) DT-MPIO binding. The amount of DT-MPIO binding detected in R1, R2, and R3 plaques of RCCA was significantly higher in the non-treated group than in the statin-treated group (p** < 0.01). Expression of inflammation biomarkers (C) MOMA-2, (D) VCAM-1, and (E) P-selectin (CD62P). Significantly higher levels of all three biomarkers were found in R1, R2, and R3 plaques of RCCA in the non-treated group as compared to the statin-treated group. Individual *p* values were indicated in Fig. 4C, D, E. (F) “Destabilising” lipid content. The amount of lipid content, measured by oil red O expression was significantly higher in R1, R2, and R3 plaques of RCCA in the non-treated group than in the statin-treated group (p** < 0.01). (G) “Stabilising” SMC content. The amount of smooth muscle cells content was significantly higher in R2, R3, and R5 plaques of RCCA in the non-treated group than in the statin-treated group (p** < 0.01). (H) Plaque vulnerability index. The vulnerability index of R1, R2, and R3 plaques of RCCA in the statin-treated group was significantly lower compared to those in the non-treated group (p** < 0.01). A high vulnerability index indicates a more vulnerable and unstable plaque

In the DT-MPIO non-treated group, substantial quantity of fluorescent-tagged DT-MPIO was detected in R1 and R2 plaques of RCCA (Fig. 5A, Fig. 6B). These plaques display high-risk inflamed plaque phenotype (i.e. increased level of inflammation biomarkers: MOMA-2, VCAM-1, P-selectin, large “destabilising” lipid burden, low “stabilising” smooth muscle cells (SMC) content in the intima) (Fig. 5A, Fig. 6). Small quantity of DT-MPIO binding was detected in R3 plaques with moderate level of inflammation and lipid burden. Only minimal DT-MPIO bound to R5 plaques (Fig. 5A, Fig. 6B). These plaques bear fairly stable and less inflamed phenotype (i.e. reduced level of inflammation biomarkers: MOMA-2, VCAM-1, P-selectin, small lipid burden, and higher SMCs content) (Fig. 5A, Fig. 6). Absence of DT-MPIO was confirmed in athero-protective zones of RCCA (R4) as well as control LCCA. The histological data confirmed the specific targeting of DT-MPIO at the high-risk inflamed plaques, discriminating them from the benign, less inflamed plaques. These results further affirmed that the conspicuous dark signal detected in R1 and R2 of RCCA in the post-contrast MRA (Figs. 2A, 3A–C, and 5A) was caused by the binding of DT-MPIO to the vulnerable plaques in the corresponding sections (Fig. 5A). Negligible amount of DT-MPIO in the histology corroborated the absence of new dark signal in the matching post-contrast images in (i) the stable, non-inflamed plaques in R5, (ii) the athero-protective R4 region of RCCA, and (iii) the control LCCA (Figs. 2A, 3A–C, and 5A).

By contrast, in the statin-treated group, the quantity of DT-MPIO binding was significantly lower in R1, R2, and R3 plaques than that in the non-treated group (Figs. 5B and 6B). Similarly, the level of inflammation biomarkers (i.e. MOMA-2, VCAM-1, and P-selectin) and the “destabilising” lipid content were also significantly reduced in R1, R2, and R3 plaques, in comparison with those in the non-treated group (Figs. 5B and 6C–F). The “stabilising” SMC amount, however, was significantly reduced in the plaques in R2, R3, and R5 in contrast with the non-treated group (Figs. 5B and 6G). Overall, the plaques in R1, R2, and R3 were substantially stabilised by statin treatment with significant reduction in the vulnerability index in these plaques (Figs. 5B and 6H). The results confirmed that absence of new discrete dark signal in R1–5 of post-contrast MR images in the statin-treated group (Figs. 2B, 3, 5B, and 6A) was consistent with the lack of DT-MPIO binding to the plaques in all regions (Figs. 5B and 6B).

In both IgG-MPIO statin-treated and non-treated groups, absent or minimal non-specific IgG-MPIO binding was detected in R1–5 plaques of whole RCCA and LCCA (Supplementary Fig. 4). The findings were in agreement with the lack of new discrete dark signal in carotid arteries on corresponding post-IgG-MPIO MRA (Supplementary Fig. 2, 3 and 4).

### Quantitative Analysis of Plaque Features (AHA Classification) on Response to Statin

**Fig. 7:**
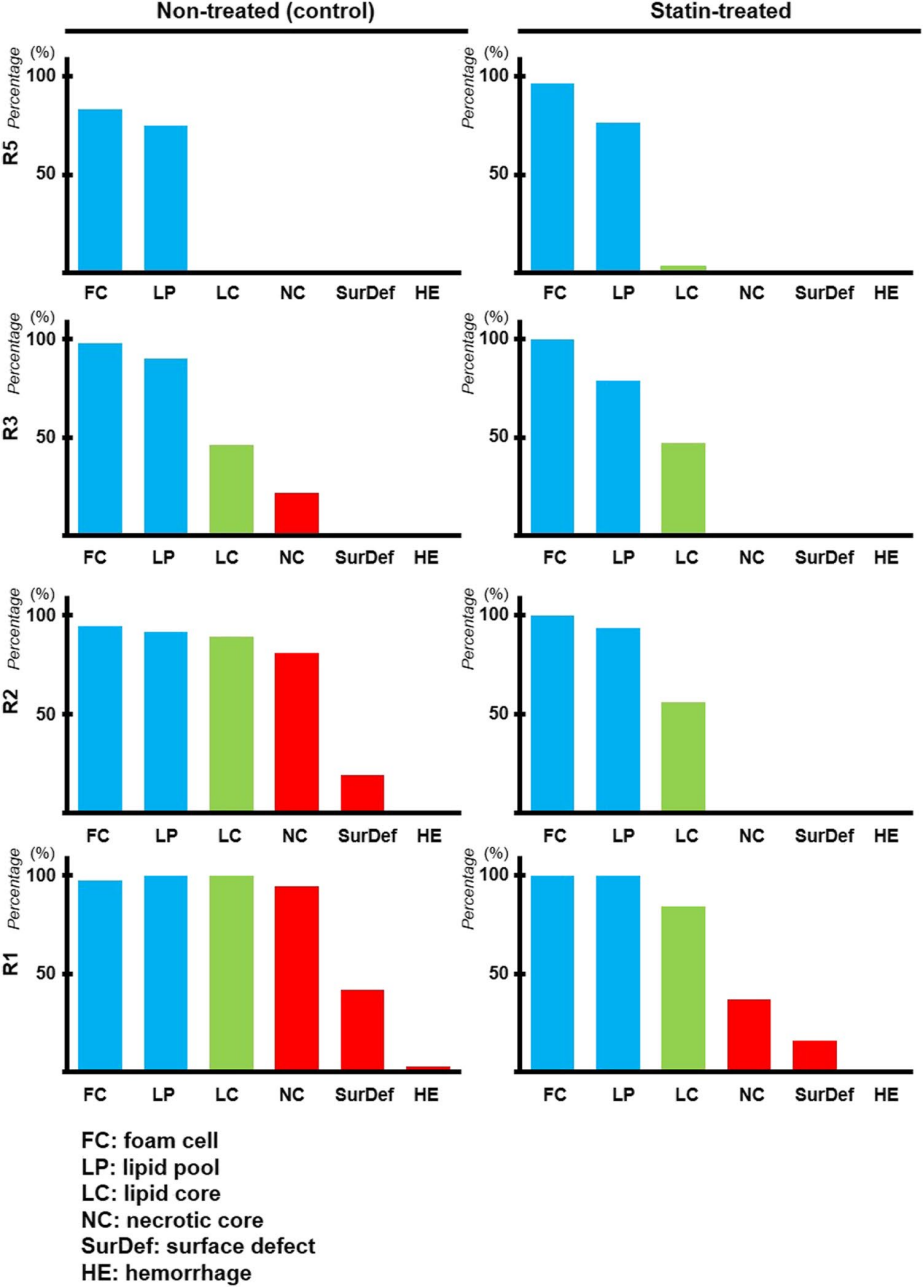
Quantitative analysis of plaque characteristics defined in the AHA classification of human plaques. Detailed results on the percentage of different plaque features detected in plaques across all regions of RCCA in both non-treated and statin-treated groups

**Fig. 8:**
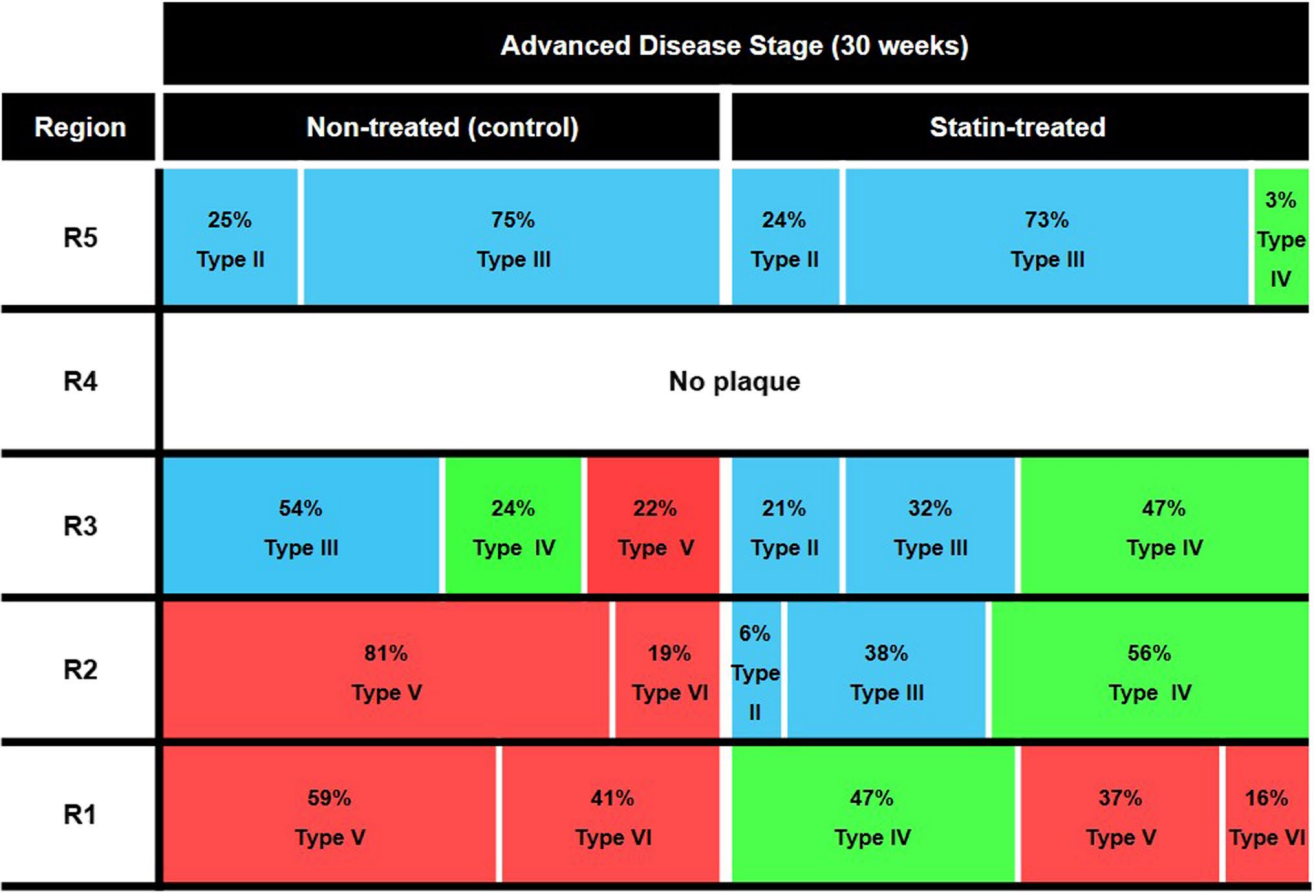
Summary of AHA classification–defined plaque features on response to statin therapy. The plaques in all regions (R1–R5) of RCCA for both statin-treated and non-treated groups were classified using the characteristics described in the AHA classification of human plaques [39, 40]

All R1 to R5 plaques in both statin-treated and non-treated groups underwent quantitative analysis using the parameters defined in the AHA classification on vascular lesions (i.e. foam cells in type 2; lipid pools in type 3; lipid core in type 4; necrotic core in type 5; surface defect and haemorrhage in type 6) (Figs. 7 and 8) [39, 40].

In the non-treated group, all sectioned samples in R1 and R2 showed high-risk, vulnerable plaques with type 5 and/or type 6 features. In R1, 41% of sections revealed type 6 complex plaques with surface defects; 2% in particular also showed intraplaque haemorrhage. Substantial portions of these complex plaques displayed necrotic cores as well. The remaining 59% sections in R1 demonstrated type 5 plaques with necrotic cores. In R2, 19% of sections revealed type 6 plaques with surface defects, lipid cores, and lipid pools. The remaining 81% sections displayed type 5 plaque features. All R3 samples were a conglomeration of vulnerable plaques with necrotic cores and/or lipid cores (~ 46% types 4 and 5) or stable plaques with lipid pools (~ 54% type 3). All R5 sections showed stable plaques with foam cells (type 2) and lipid pools (type 3).

By contrast, in the statin-treated group, 16% of sections revealed type 6 plaques with surface defects while 37% sections showed type 5 plaques with necrotic cores, lipid cores, and lipid pools in R1. The remaining 47% sections displayed type 4 plaques with lipid cores. In R2, 56% of the sections displayed advanced plaques with lipid cores (type 4) and 44% showed stable plaques with foam cells (type 2) and lipid pools (type 3). In R3, 47% revealed type 4 advanced plaques with lipid cores and lipid pools while 53% showed stable plaques with type 2 and type 3 features. In R5, almost all Sects. (97%) displayed stable plaques with type 2 and type 3 features. Only 3% exhibited type 4 plaques with lipid cores.

## Discussion

There is active debate in managing carotid disease in asymptomatic patients. Annual stroke risk in asymptomatic patients on medical therapy has lowered to 0.5–1% [41]. Nevertheless, even on best medical therapy, about 10–15% of asymptomatic patients still have increased risk for stroke [42]. Hence, this patient subgroup is the one that truly benefits from carotid stenting or endarterectomy. The challenge is to (i) accurately monitor the risk of carotid plaques to ensure the medical therapy instituted is indeed effective; and if not, (ii) timely identify these high-risk patients for prophylactic carotid intervention, proactively preventing stroke [41, 42].

Herein, we have reported on the development of a DT-MPIO-enhanced MR imaging tool to (i) identify and characterise the high-risk vulnerable plaques; (ii) quantitatively track the inflammatory status of plaque development; and (iii) monitor the response to statin therapy longitudinally. This is the first pre-clinical study to use molecular an MRI tool to monitor response to atheroma pharmacotherapy longitudinally in the cuff implantation progressive atherosclerosis model. Furthermore, it is also the first pre-clinical study to utilise AHA classification of human plaques, a clinically relevant parameter, to evaluate molecular imaging–defined therapeutic response in atherosclerosis, approximating the clinical translation of this molecular imaging tool.

Molecular MR imaging tools have been previously reported to interrogate the underlying inflammatory activities within atherosclerotic plaques in pre-clinical and clinical studies [7, 25–27]. Amongst these, non-targeted ultra-small superparamagnetic iron oxide (USPIO)–enhanced MRI were utilised to identify macrophage-rich plaques in patients with both symptomatic and contralateral asymptomatic carotid disease [27]. In the ATHEROMA study (Atorvastatin Therapy: Effects on Reduction of Macrophage Activity), this molecular MRI tool was used to demonstrate the anti-inflammatory effects of high dose statin with marked decrease in USPIO uptake in patient carotid plaques [7]. However, the extended blood circulation time of USPIO prolonged the background blood phase contrast, hence increasing the time-lag between USPIO injection and image acquisition, impeding the widespread application in acute clinical conditions. The typical intervals between USPIO injection and imaging, in humans and small animals, are 24–36 h [7, 43] and 2–4 days [44, 45], respectively, although active targeting strategies in pre-clinical studies have reduced this interval to between 8 and 24 h [46, 47]. Furthermore, the plaque macrophage populations, which are in constant flux, are hence sensitive to temporal changes [48]. In the emergency of stroke management, the extended imaging time-lag may hamper the differentiation of the observed signal between plaque instability preluding the symptoms or the after-effects of cerebrovascular event.

Compared with passive uptake of USPIO by plaque macrophages, active binding of ligand-conjugated MPIO to molecular targets may enhance the utility of imaging the acute inflammatory processes in atherosclerotic disease [32, 49, 50] and ischaemic-reperfusion injury [51]. The advantages of DT-MPIO-enhanced imaging in acute clinical setting were further illustrated in this study, whereby (i) a higher iron payload of MPIO markedly amplifies the contrast effects to enable in vivo detection and monitoring of inflammatory biomarkers within the plaques; (ii) DT-MPIO has faster blood clearance than USPIO, with a half-life of 1.75 min versus 11 h respectively [32, 46]. This reduces the background of blood phase contrast, enhancing signal detection in plaques; (iii) DT-MPIO are designed to mimic the firm adhesion and rolling action of circulating monocytes to the vessel wall, mediated by VCAM-1 and P-selectin. Exploiting the rapid antigen–antibody interaction, DT-MPIO-induced MR contrast effects were detected promptly from 30 min and sustained up to 2 h post-injection [32, 34], a feasible imaging period for assessing acute cerebrovascular events.

Our previous studies have used a cuff implantation mouse model to develop a robust testing platform that generates distinct stages of human-like atherosclerotic plaques along a single carotid artery [34, 35]. The competitive advantages of this platform were capitalised in this study, with (i) high clinical translatability — the model was systematically examined using clinically relevant features, including inflammation, vulnerability, risk levels, and the gold standard AHA classification of human plaques [39] as benchmark for clinical translation. (ii) Near 100% accuracy in predicting exactly where (anatomical locations R1–5), when (duration after cuff implantation), and what (AHA types/stages/risks) plaques will develop. (iii) Single artery model — this model can produce low-/medium-/high-risk human-like plaques in distinct locations along a single carotid artery, enabling objective evaluation of targeting capabilities of molecular probes, without local haemodynamic and inter-subject variability. (iv) Predictive progressive translational model — the plaques in this model have been generated in a predictive manner, longitudinally followed up, and evaluated using clinically relevant parameters from early, intermediate, to advanced disease stage. Moreover, in ApoE^−/−^ mice, their blood cholesterol levels are reportedly not affected by statins [52], since they lack ApoE [53, 54], the major ligand for the low-density lipoprotein (LDL) receptor. Statins are also swiftly metabolised in these mice’s livers [55]. These factors collectively make the ApoE^−/−^ mouse an ideal model to investigate exclusively the anti-inflammatory effects of 3-hydroxy-3-methyl-glutaryl-CoA reductase (HMGR) inhibition.

Exploiting this model, we have shown that DT-MPIO-enhanced MRI can identify high-risk vulnerable plaques, discriminate the heterogeneousness within the asymptomatic carotid plaque population, and quantitatively report the inflammatory and vulnerability status of local plaques, thus achieving in vivo characterisation and risk stratification of carotid atherosclerosis (i.e. prominent MR signal in high-risk vulnerable plaques in R1 and R2, modest signal in medium-risk plaques in R3, and negligible signal in low-risk stable plaques in R5). In support of these observations, we have previously used dual-targeted iron particles enhanced MRI to characterise inflammation and plaque vulnerability across a range of models: (i) endothelial cell inflammation model [56], (ii) patient carotid plaques [31], (iii) aortic root plaques in ApoE^−/−^ mouse model [32], and (iv) carotid plaques of low/medium/high-risk levels in cuff implantation mouse model [32, 34]. By directly reporting the inflammatory status of local plaques, DT-MPIO-enhanced MRI may overcome the limitations of current angiographic techniques that could miss the detection of vulnerable plaques due to positive vascular remodelling [57, 58].

In addition, DT-MPIO-enhanced MRI is capable of quantitatively tracking plaque inflammation and vulnerability in all regions of RCCA longitudinally, proportionate to the degree of hypointense MRI signals, throughout atherosclerosis progression in the non-treated group. This is consistent with our progressive atherosclerosis model, where high-, medium-, and low-risk plaques were generated in R1/2, R3, and R5 of RCCA, respectively, with increasing degree of inflammation and vulnerability index, from early to intermediate and advanced disease stage (15, 20, and 30 weeks post-cuff implantation) [35]. Moreover, this molecular imaging tool can monitor the response to statin therapy, whereby demonstrating that plaque inflammation and vulnerability index in R1, R2, and R3 of RCCA were significantly attenuated in the statin-treated group, compared with the non-treated group. Importantly, this molecular imaging–defined therapeutic response in atherosclerosis was further validated by the gold standard clinically relevant parameter — the AHA classification of human plaques. Statin therapy has reversed the plaque progression from (i) 100% of high-risk complicated plaques (AHA type 5/6) to 53% only in R1, (ii) 100% of high-risk plaques (type 5/6) to 100% of low- and medium-risk plaques (type 2/3/4) in R2, and (iii) 100% of medium- and high-risk plaques (type 3/4/5) to 100% of low- and medium-risk plaques (type 2/3/4) in R3. The superior contrast sensitivity, fast targeting, and blood clearance of DT-MPIO promote a prominent and quantifiable signal effect that enables real-time in vivo monitoring of therapeutic response in clinically relevant parameters in atherosclerosis, moving a step closer to clinical development of this molecular imaging tool.

Ferumoxytol iron-oxide particles have displayed favourable safety profile and are clinically approved therapeutic agents for patients with iron deficiency anaemia [59]. Additionally, these ferrous-based contrast agents were utilised in clinical MRI of vascular conditions and atherosclerosis [7, 60, 61], and as a viable alternative for 20–40% vascular patients with chronic renal disease, who are prone to risks of renal failure associated with conventional gadolinium- and iodine-based contrast agents [60, 61].

Limitations of our study include the moderately high dose of MPIO (30 mg Fe/kg body weight) utilised to attain quantitative reporting and tracking of the inflammatory status within plaques. This dose was higher than that used in clinical atherosclerosis imaging studies (3–4 mg Fe/kg) [60, 61] but lower than the dose administered in pre-clinical studies involving small animals (10–56 mg Fe/kg) [45–47, 62]. Further optimisation of the dose of MPIO is required. Although ApoE^−/−^ mice were specifically chosen to investigate exclusively the anti-inflammatory effects of HMGR inhibition, serum lipid profile could have been included in this study to provide an additional reference when comparing between the statin group and non-treatment group. In addition, this molecular imaging tool was developed in a preclinical mouse model. Further development and validation of this tool in large animal models is required prior to translation into clinical arena. Future work also includes exploration of advanced MRI techniques, such as susceptibility-weighted imaging, that could potentially increase the sensitivity for in vivo detection of the iron molecular probes.

In summary, in vivo DT-MPIO-enhanced MRI can identify high-risk vulnerable plaques, stratify the risk within the asymptomatic plaque population, and accurately monitor the inflammatory status and risk of carotid plaques to ensure the statin therapy instituted is indeed effective. With further development and translation into clinical arena, this molecular imaging strategy may permit timely identification of the high-risk asymptomatic patients, who are unresponsive to best medical therapy, expediting prophylactic carotid intervention for stroke prevention, facilitating personalised management of carotid atherosclerotic disease. This molecular imaging strategy may aid in decision-making for launching major clinical endpoint trials by refining trial population and dose selection, potentially expediting new cardiovascular therapeutics to the market.

### Supplementary Information

Below is the link to the electronic supplementary material.

Supplementary file1 (DOCX 3516 KB)

## Data Availability

The datasets generated during and/or analysed during the current study are available from the corresponding author on reasonable request.

## Abbreviations

^18^F-FDG PET: ^18^F-fluorodeoxyglucose positron emission tomography
ACST: Asymptomatic Carotid Surgery Trial
ACT I: Asymptomatic Carotid Trial
AHA: American Heart Association
ApoE^−/−^: Apolipoprotein E–deficient mice, ApoE knock-out mice
ATHEROMA: Atorvastatin Therapy: Effects on Reduction of Macrophage Activity
AUC: Area under curve
CD62P: P-selectin
CEUS: Contrast-enhanced ultrasound
CREST: Carotid Revascularization Endarterectomy versus Stenting Trial
CT: Computed tomography
DT-MPIO: Dual-targeted microparticles of iron oxide
FDA: Food and Drug Administration
FLASH: Fast low-angle shot
FOV: Field-of-view
HMGR: 3-Hydroxy-3-methyl-glutaryl-CoA reductase
HSS: High shear stress
LCCA: Left common carotid artery
LDL: Low-density lipoprotein
LSS: Low shear stress
MOMA-2: Anti-macrophages/monocytes antibody
MRI: Magnetic resonance imaging
OSS: Oscillatory shear stress
RCCA: Right common carotid artery
SMA: Smooth muscle alpha-actin
SMC: Smooth muscle cells
TE: Echo time
TOF MRA: Time-of-fight angiography
TR: Repetition time
USPIO: Ultrasmall superparamagnetic iron oxide
VCAM-1: Vascular cell adhesion molecule-1
