# Supplementary material for: Translational Molecular Imaging Tool of Vulnerable Carotid Plaque: Evaluate Effects of Statin Therapy on Plaque Inflammation and American Heart Association–Defined Risk Levels in Cuff-Implanted Apolipoprotein E–Deficient Mice
Source: Transl Stroke Res. 2022 Dec 9;15(1):110–26. doi: 10.1007/s12975-022-01114-4 (PMC10796420; doi:10.1007/s12975-022-01114-4)
Supplement: Supplementary file 1 — Supplementary file1 (DOCX 3516 KB) [file 12975_2022_1114_MOESM1_ESM.docx]

**Supplementary Information for**

**Translational molecular imaging tool of vulnerable carotid plaque: evaluate effects of statin therapy on plaque inflammation and American Heart Association-defined risk levels in cuff-implanted Apolipoprotein E-deficient mice**

^*^Joyce MS Chan MBBS PhD^1,2,3^, Sung-Jin Park PhD^1^, Michael Ng BSc^1^, Way Cherng Chen PhD^4^, Wan Ying Chan MBBS FRCR^5^, Kishore Bhakoo PhD^6^, Tze Tec Chong MBBS FACS^2^

^1^Translational Cardiovascular Imaging Group, Institute of Bioengineering and Bioimaging (IBB), Agency for Science, Technology and Research (A*STAR), 11 Biopolis Way, #02-02 Helios, Singapore 138667

^2^Department of Vascular Surgery, Singapore General Hospital, SingHealth, Outram Road, Singapore 169608

^3^Lee Kong Chian School of Medicine, Nanyang Technological University, 50 Nanyang Avenue, Singapore 639798

^4^Bruker Singapore Pte. Ltd., 30 Biopolis Street, Matrix #09-01, Singapore 138671

^5^Division of Oncologic Imaging, National Cancer Centre, Singapore

^6^Institute of Bioengineering and Bioimaging (IBB), Agency for Science, Technology and Research (A*STAR), 11 Biopolis Way, #02-02 Helios, Singapore 138667

**List of Supplementary Methods:**

1. **Supplementary Table 1**. Primary antibody information
2. **Supplementary Table 2**. Secondary antibody information

**List of Supplementary Figures:**

1. **Supplementary Figure 1.** Simplified diagram to illustrate the quantification of MPIO-induced hypointense signal by the difference in AUC
2. **Supplementary Figure 2.** Serial *in-vivo* IgG-MPIO enhanced MRA of carotid arteries to monitor response to statin treatment longitudinally
3. **Supplementary Figure 3.** Quantification of IgG-MPIO induced MR signal change in statin-treated and non-treated groups
4. **Supplementary Figure 4.** Histological analysis of all regions in RCCA in IgG-MPIO statin-treated group and non-treated group

**Supplementary Methods**

**Synthesis of fluorescent-tagged dual antibody-conjugated MPIO**

In a single vessel, add 1μm-sized iron oxide particles (Dynabeads™ MyOne™ Tosylactivated; Invitrogen, 20 mg), fluorescein cadaverine (LifeTech, Singapore), purified monoclonal rat anti-mouse antibodies for VCAM-1 (CD106; BD Pharmingen™; Zuellig Pharma, 40 µg) and P-selectin (CD62P; Santa Cruz Biotechnology; Axil Scientific, 40 µg). Leave the mixture on slow tilt rotation at 37°C for 20 hours. After 20 hours incubation, wash the MPIO mixture twice with cold wash/store buffer (Phosphate buffered saline (PBS) pH 7.4, 0.1% bovine serum albumin (BSA), 0.05% Tween® 20) and further mix with blocking buffer (PBS pH 7.4, 0.5% BSA, 0.05% Tween® 20) at 37°C overnight. Lastly, wash mixture 3 times with cold wash/store buffer for 5 minutes per wash and store DT-MPIO at 4°C in PBS with 0.1% BSA (40 mg Fe/ mL). IgG-MPIO was made by conjugating the purified monoclonal rat anti-mouse antibodies for IgG-1 (AbD Serotec; SciMed (Asia) Pte Ltd; Singapore, 80 µg per 20 mg iron oxide particles) and fluorescein cadaverine to the iron particles and used as a control to evaluate the non-specific binding of particles to the plaques.

**Histology and immunohistochemical staining of carotid arteries**

**Tissue sample preparation**

All four groups of animals (DT-MPIO statin-treated, DT-MPIO non-treated, control IgG-MPIO statin-treated, control IgG-MPIO non-treated) were euthanized at the end of their week 30 serial *in vivo* MRI scan for histology analysis. The RCCA and LCCA of each animal was surgically harvested and paraformaldehyde (4% in PBS solution) was used to fix them at 4°C for 2 hours. Next, PBS was used to wash the samples, followed by dehydration using sucrose (30% in PBS). Lastly, optimal cutting temperature (OCT) compound was used to freeze the samples before serially sectioning them at 12 µm thickness on a cryostat (Leica CM1950®).

**Haematoxylin and eosin (H & E) stain**

Sectioned samples were rinsed with PBS to remove residual OCT compounds. Samples were incubated with Haematoxylin solution Gill No.3 (Sigma-Aldrich®, GHS316) for 5 minutes and washed with distilled water for 10 minutes to induce metachromatic stain. Next, Eosin Y solution (Sigma-Aldrich®, HT10132) was added for 1 minute and briefly rinsed with distilled water. Samples were treated with 70, 80, 90, 100% ethanol and 100% xylene to dehydrate the tissue. Lastly, the samples were mounted with a cover glass using Canada Balsam mounting media.

**Oil red O (ORO) stain**

Sectioned samples were rinsed with distilled water followed by 60% isopropanol (IPA). Freshly prepared ORO solution (0.3g ORO, 60 mL IPA, 40 mL distilled water) was added to samples for 15 minutes followed by a brief wash with 60% IPA. PBS was used to wash the samples before mounting them onto microscope slides (ProLong^TM^ Gold Antifade Mountant with DAPI, ThermoFisher Scientific).

**Immunohistochemistry**

Sectioned samples were rinsed with PBS to remove residual OCT compounds. Samples were treated with 1% BSA for 30 minutes to block non-specific binding. Dual primary antibodies of different hosts (Supplementary Table 1) were added to the samples to be incubated at 4°C overnight. After which, samples were washed with PBS followed by treatment with secondary antibodies (Supplementary Table 2) for 2 hours at room temperature. Lastly, PBS was used to wash the samples before mounting them onto microscope slides (ProLong^TM^ Gold Antifade Mountant with DAPI, ThermoFisher Scientific).

**Supplementary Table 1.** Primary antibody information.

|  | Primary antibody | Host | Company | Cat. No. | Dilution factor |
| --- | --- | --- | --- | --- | --- |
| **4 basic antibodies** | Monocyte + macrophage  (MOMA-2) | Rat | Abcam | Ab33451 | 1:100 |
|  | Alpha smooth muscle actin (SMA) | Goat | Abcam | Ab21027 | 1:200 |
|  | CD62P (p-selectin) | Mouse | Abcam | Ab54427 | 1:100 |
|  | Vascular cell adhesion protein 1  (VCAM-1) | Rabbit | Abcam | Ab134047 | 1:100 |

**Supplementary Table 2.** Secondary antibody information.

|  | Secondary antibody | Host | Company | Cat. No. | Dilution factor |
| --- | --- | --- | --- | --- | --- |
| **Fluorescent conjugated** | AlexaFluore^®^ 594 conjugated Rat IgG H&L | Donkey | Abcam | Ab150156 | 1:4000 |
|  | AlexaFluore^®^ 647 conjugated Goat IgG H&L | Donkey | Abcam | Ab150135 | 1:4000 |
|  | AlexaFluore^®^ 594 conjugated Mouse IgG H&L | Donkey | Abcam | Ab150112 | 1:5000 |
|  | AlexaFluore^®^ 647 conjugated Rabbit IgG H&L | Donkey | Abcam | Ab150063 | 1:4000 |


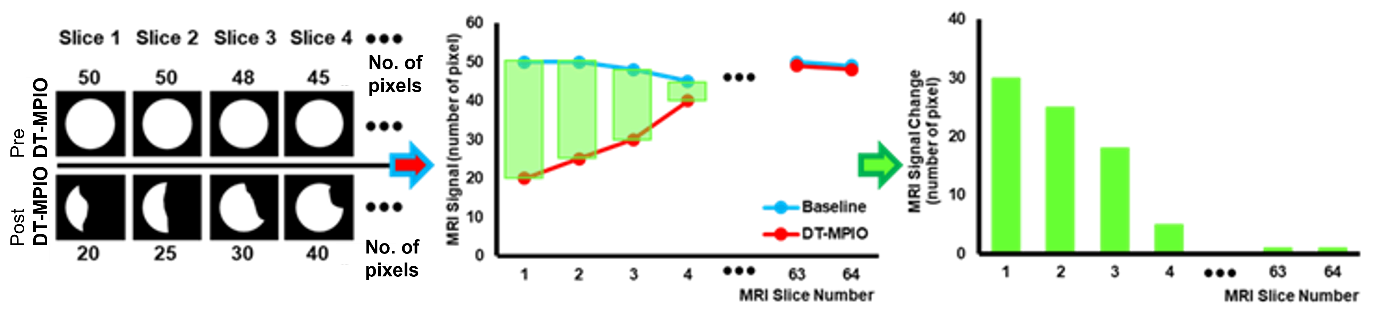


**Supplementary Figure 1.** Simplified diagram to illustrate the quantification of MPIO-induced hypointense signal by the difference in AUC


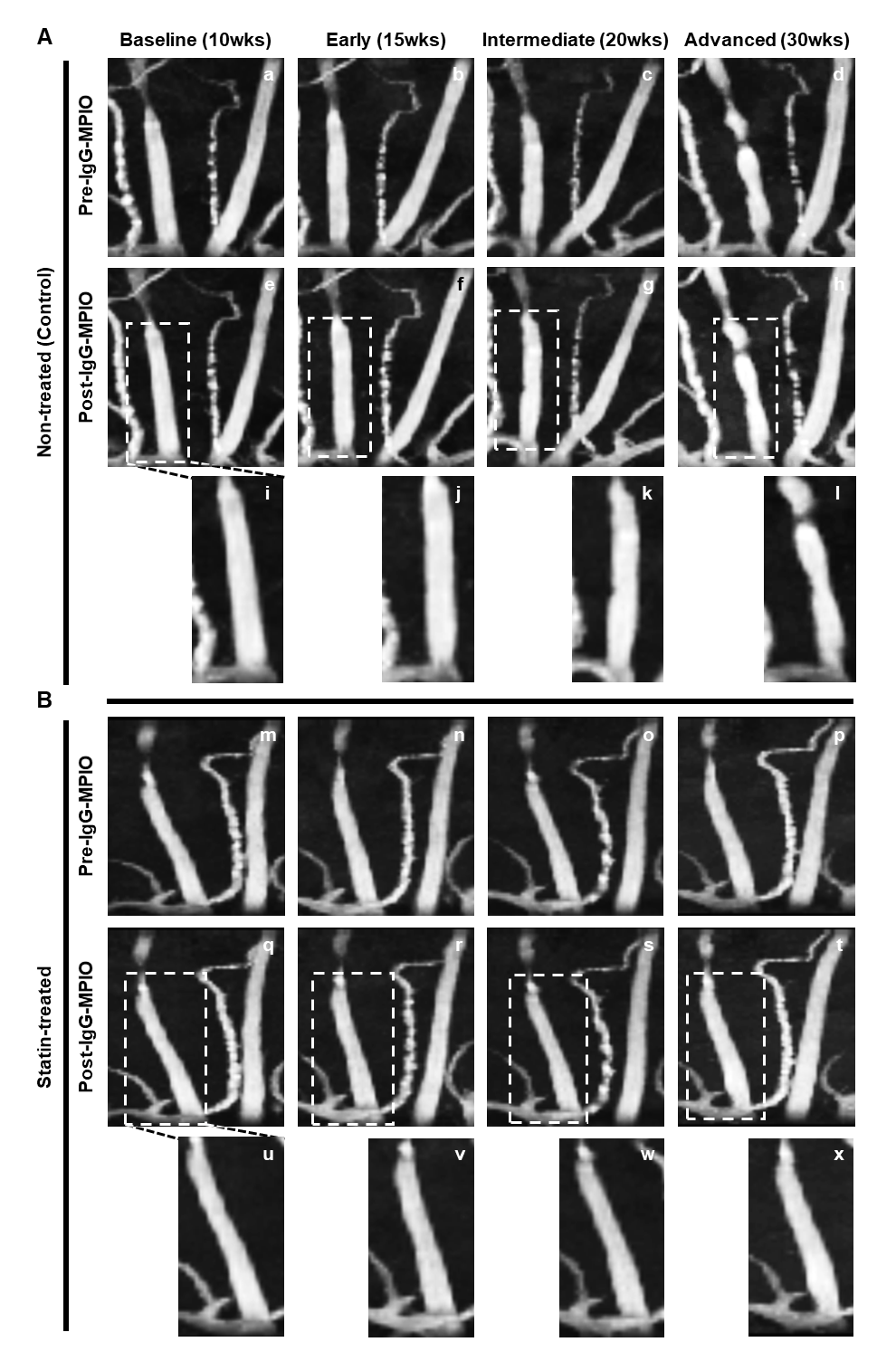


**Supplementary Figure 2.** **Serial *in-vivo­* IgG-MPIO enhanced MRA of carotid arteries to monitor response to statin treatment longitudinally. (A). IgG-MPIO non-treated group. (a-d).** Pre-IgG-MPIO MRA images of carotid arteries as atherosclerosis progresses from baseline (10 weeks) to early (15 weeks), intermediate (20weeks) and advanced (30 weeks) stage. **(e-h).** Post-IgG-MPIO MRA images. **(i-l).** Magnified MRA images of post-IgG-MPIO RCCA. No new dark signal was identified in all stages. **(B). IgG-MPIO statin-treated group. (m-p).** Pre-IgG-MPIO MRA images of carotid arteries as atherosclerosis progresses from baseline to early, intermediate and advanced stage. **(q-t).** Post-DT-MPIO MRA images. **(u-x).** Magnified MRA images of post-DT-MPIO RCCA. No new dark signal was observed in baseline before commencement of statin and in all stages of atherosclerosis disease.


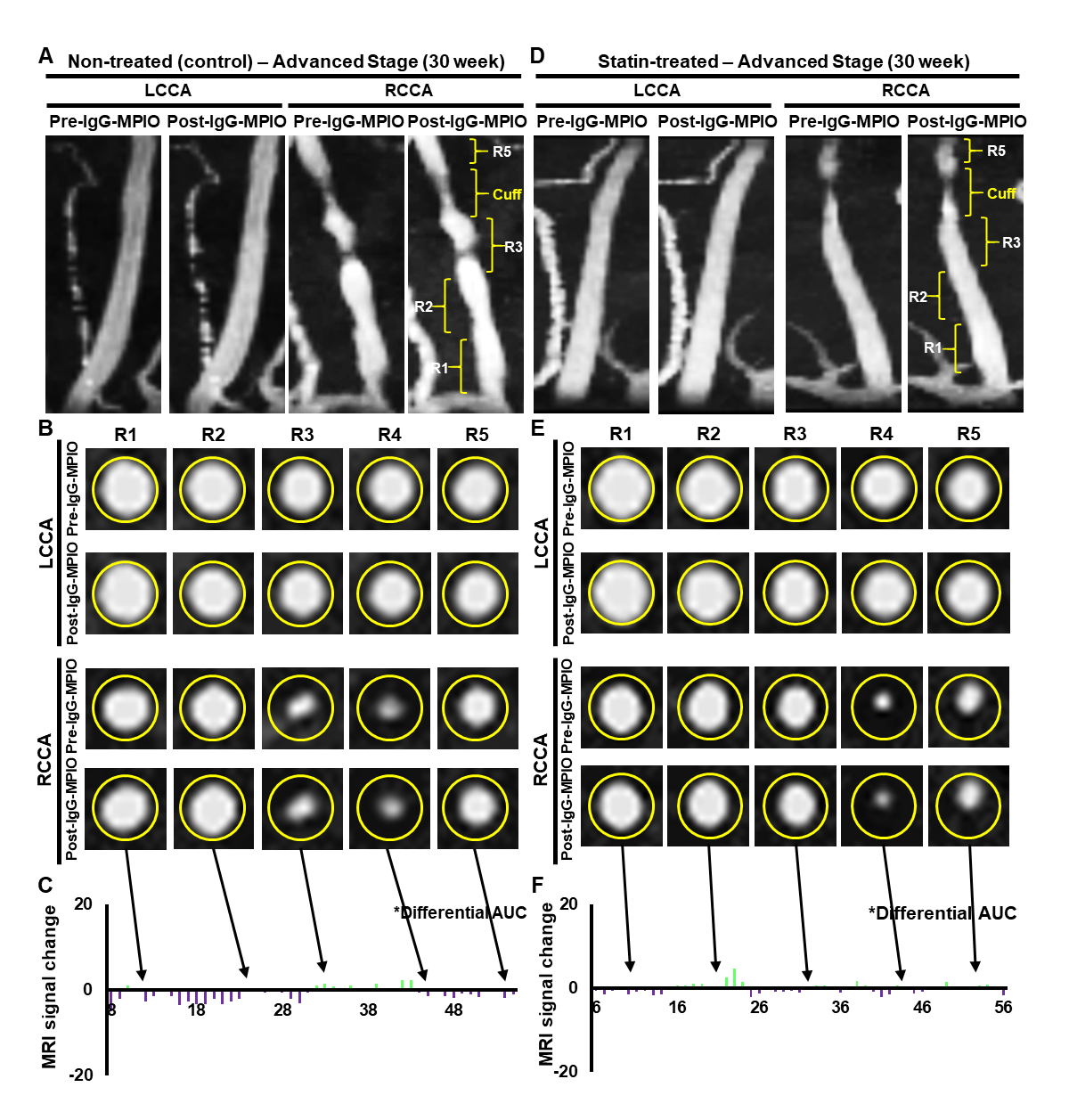


**Supplementary Figure 3. Quantification of IgG-MPIO induced MR signal change in statin-treated and non-treated groups. (A). Representative IgG-MPIO-enhanced TOF-MRA images of carotid arteries in non-treated group at 30 weeks.** No new discrete dark signals detected in post-contrast images of both carotid arteries. **(B). Corresponding transverse plane MR images of LCCA and RCCA in non-treated group.** No new discrete dark signals detected on the matching post-IgG-MPIO transverse planes in RCCA. **(C). Quantification of MRI signal change between pre- and post- contrast MR images of carotid arteries.** The degree of change in MR signal was minimal in the whole RCCA. **(D). Representative IgG-MPIO-enhanced TOF-MRA images of carotid arteries in statin-treated group at 30 weeks.** No new dark signal detected in post-contrast images of both carotid arteries. **(E). Corresponding transverse plane MR images of LCCA and RCCA in statin-treated group.** No new discrete dark signal detected on the matching post-DT-MPIO transverse planes in both carotid arteries. **(F). Quantification of MRI signal change between pre- and post- contrast MR images of carotid arteries.** The degree of change in signal was minimal in the whole RCCA.

**
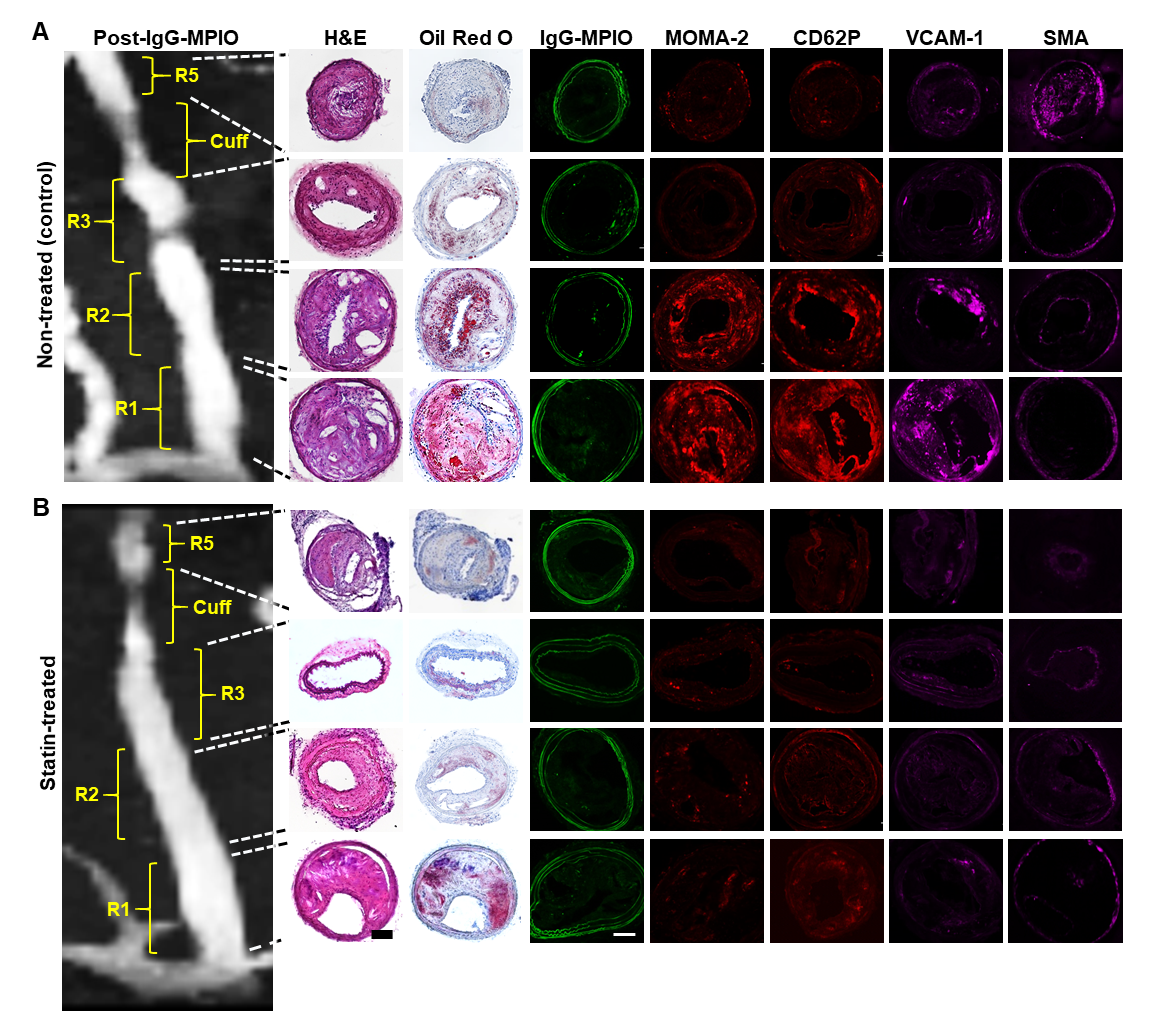
**

**Supplementary Figure 4.** **Histological analysis of all regions in RCCA in IgG-MPIO statin-treated group and non-treated group. (A). Representative post-IgG-MPIO MRA image of RCCA in non-treated group with matching histological sections.** Minimal quantity of fluorescent-tagged IgG-MPIO was observed in R1, R2, R3 and R5 plaques. The R1 and R2 plaques display high-risk inflamed plaque phenotype [i.e. increased level of inflammation biomarkers: MOMA-2, P-selectin, VCAM-1, large “destabilising” lipid burden (Oil Red O), low “stabilising” smooth muscle cells (SMC) content in the intima]. R3 plaques display moderate levels of inflammation and lipid burden. R5 plaques display fairly stable and less inflamed phenotype [i.e. reduced level of inflammation biomarkers: MOMA-2, P-selectin, VCAM-1, small lipid burden and higher SMCs content)]. **(B). Representative post-IgG-MPIO MRA image of RCCA in statin-treated group with matching histological sections.** Minimal amount of IgG-MPIO was observed in R1, R2, R3 and R5 plaques. The plaques display reduced levels of inflammation biomarkers (i.e. MOMA-2, P-selectin, VCAM-1) and lower level of “destabilising” lipid content as compared to those in the corresponding regions in the non-treated group. However, the “stabilising” SMC amount in R3 and R5 plaques were also lower than those in the non-treated group.
